# PW01-015 – Canakinumab in adults with colchicin resistant FMF

**DOI:** 10.1186/1546-0096-11-S1-A68

**Published:** 2013-11-08

**Authors:** A Gül, H Özdoğan, Ö Kasapçopur, B Erer, S Uğurlu, N Davis, S Sevgi

**Affiliations:** 1 Istanbul Faculty of Medicine, Turkey; 2 Cerrahpaşa Faculty of Medicine, Istanbul University, İstanbul, Turkey; 3 Novartis, East Hannover, NJ, USA; 4 Novartis, İstanbul, Turkey

## Introduction

Familial Mediterranean fever (FMF) is associated with variations in the MEFV gene resulting in proteolytic activation of IL-1β through the inflammasome complex. There is no established treatment available for those resistant or intolerant to standard of care colchicine treatment. Canakinumab, a fully human selective anti-IL-1β monoclonal antibody with a half-life of ~4-weeks binds to human IL-1β and neutralizes its proinflammatory effects.

## Objectives

To evaluate the efficacy and safety of canakinumab in adolescents and adults with FMF who are resistant or intolerant to colchicine.

## Methods

FMF patients with ≥1 attack/month in the preceding 3-months despite the highest tolerated colchicine dose were eligible to enter a 30-day run-in period. Those with an attack in the run-in period advanced to a 3-month treatment period to receive canakinumab 150mg sc every 4-weeks starting at the next attack in the following month. Patients then followed-up for up to 2 months or until the next attack. Attacks were confirmed by presence of fever, serositis, and elevated CRP. Primary efficacy outcome was the proportion of patients with ≥50% reduction in time-adjusted attack frequency in the treatment vs pre-treatment periods. Secondary objectives included the percent of patients with no attacks in the treatment period, time to next attack after the last canakinumab dose, and changes in the quality of life by SF-36. Safety was assessed by AEs and laboratory values at each visit.

## Results

Thirteen patients enrolled in the *run-in* and 9 (median age 22 yrs, range 12-34 yrs) entered *treatment* periods. Only 1 patient had an attack (peritonitis) during the treatment period and all had a ≥50% reduction in their time-adjusted pre-treatment attack rate. Median baseline elevated CRP (58mg/L) and serum AA (162mg/L) levels normalized (CRP, 2.5mg/L; SAA, 5.8mg/L) by Day 8 and remained low throughout the study. The Physical and Mental Component scores of the SF-36 improved from a median baseline of 32 and 38 to 81 and 78 at Day 8 respectively, and continued to improve throughout the treatment period. Five patients had an attack in the follow-up period, which occurred a median 71 days (31-78 days) from the last canakinumab dose. Compared to baseline, the physician and patient global assessment of FMF control improved with treatment with overall the response to treatment reported as Very Good by both physicians (9/9) and patients (7/9) at study end. Eight patients reported at least one adverse event (AE) with headache (n=4) and upper respiratory tract infection (n=2) being the only AEs reported in more than 1 patient. No one discontinued early from the trial.

## Conclusion

In this pilot study, canakinumab was found to be effective at controlling the attack recurrence in FMF patients resistant or intolerant to colchicine. AEs were similar to previous canakinumab trials and were managable. Further studies are warranted to explore the role of canakinumab in the treatment of FMF.

## Disclosure of interest

A. Gül Grant / Research Support from: Novartis, Xoma, Servier, Consultant for: Novartis, Xoma, H. Özdoğan Grant / Research Support from: Novartis, Consultant for: Novartis, Ö. Kasapçopur Grant / Research Support from: Novartis, Consultant for: Novartis, B. Erer: None Declared, S. Uğurlu: None Declared, N. Davis Employee of: Novartis, S. Sevgi Employee of: Novartis

